# Evaluation of a Fast Test to Identify the Presence of Proline Aminopeptidase in Women With Bacterial Vaginosis

**DOI:** 10.1155/S1064744997000380

**Published:** 1997

**Authors:** Ernesto Calderón, Roberto Rivera, Silvia Gordillo, Carlos Conde-Glez

**Affiliations:** 1 Obstetrics and Gynecology Clinic Hospital Juárez de México Mexico City Mexico; 2 Research Center on Infectious Diseases National Public Health Cuernavaca Morelos Mexico; 3 Rodríguez Saro N. 523 Department 104-A Col. Del Valle C.P. 03100 México D.F. Mexico

## Abstract

*Objective:* The purpose of this study was to evaluate the activity of proline aminopeptidase by a rapid paper strip test in women with bacterial vaginosis (BV).

*Methods:* Vaginal secretions of 1,387 voluntary patients attending the Obstetrics and Gynecology Infectious Diseases Clinic at Juárez Hospital of Mexico City were collected and examined. Patients were assigned into 2 groups: 483 with BV according to clinical and laboratory criteria and 604 without BV as the control group. For the purposes of this study, 300 patients with trichomonas and/or yeast were excluded from the BV group. The strips were prepared by using L-proline β-naphthylamide and L-proline p-nitroanilide as the substrates to detect proline aminopeptidase activity in concentrated vaginal secretions. In parallel, all samples were also analyzed with the standard methods in microplates containing either sustrate as a control of the rapid strip test. The test was interpreted after 3–5 min of incubation.

*Results:* The results in the strip and microplate assays were similar in 95% of the samples. Sensitivity was 91.7% and specificity was 94.2%; probability of BV if the test is positive was 92.6% and negative predictive value was 93.4%.

*Conclusions:* These findings indicate that this aminopeptidase rapid strip assay provides a 3–5 min identification of activity of the enzyme in women with BV. The procedure is a rapid, non-expensive, sensitive, and useful test at the gynecologic clinic.


Infectious Diseases in Obstetrics and Gynecology 5:226–231 (1997)
© 1997 Wiley-Liss, Inc.

### Evaluation of a Fast Test to Identify the Presence of Proline Aminopeptidase in Women With Bacterial Vaginosis

Ernesto Calderón,* Roberto Rivera,[1] Silvia Gordillo,[1] and Carlos Conde-Glez[2]

[1]*Obstetrics and Gynecology Clinic, Hospital Juárez de México, Mexico City, México*

[2]*Research Center on Infectious Diseases, National Public Health, Cuernavaca, Morelos, México*

ABSTRACT

*Objective:* The purpose of this study was to evaluate the activity of proline aminopeptidase by a rapid paper strip test in women with bacterial vaginosis (BV).

*Methods:* Vaginal secretions of 1,387 voluntary patients attending the Obstetrics and Gynecology Infectious Diseases Clinic at Juárez Hospital of Mexico City were collected and examined. Patients were assigned into 2 groups: 483 with BV according to clinical and laboratory criteria and 604 without BV as the control group. For the purposes of this study, 300 patients with trichomonas and/or yeast were excluded from the BV group. The strips were prepared by using L-proline β-naphthylamide and L-proline p-nitroanilide as the substrates to detect proline aminopeptidase activity in concentrated vaginal secretions. In parallel, all samples were also analyzed with the standard methods in microplates containing either sustrate as a control of the rapid strip test. The test was interpreted after 3–5 min of incubation.

*Results:* The results in the strip and microplate assays were similar in 95% of the samples. Sensitivity was 91.7% and specificity was 94.2%; probability of BV if the test is positive was 92.6% and negative predictive value was 93.4%.

*Conclusions:* These findings indicate that this aminopeptidase rapid strip assay provides a 3–5 min identification of activity of the enzyme in women with BV. The procedure is a rapid, non-expensive, sensitive, and useful test at the gynecologic clinic. Infect. Dis. Obstet. Gynecol. 5:226–231, 1997.
© 1997 Wiley-Liss, Inc.

KEY WORDS
vaginal discharge; non-specific vaginitis; rapid diagnosis; reproductive tract infection

Bacterial vaginosis (BV) is an abnormal condition of the vaginal ecosystem which, from a microbiological point of view, is characterized primarily by the replacement of dominant vaginal flora with *Gardnerella vaginalis, Bacteroides* spp., *Mobiluncus* spp., and genital *Mycoplasma.*[1–8]

BV is one of the most common infections of the genital tract occurring primarily in women with an active sex life in their reproductive years. BV has been associated with different infectious processes such as urinary infection, postpartum endometritis, intra-amniotic infection, premature labor, premature rupture of membranes, and low birth weight.[1]

Although both clinical and laboratory diagnostic procedures for BV exist,[4] they may be difficult to interpret, especially when trying to establish a diagnosis during an outpatient visit. Some of the limitations are equipment availability, experience

*Correspondence to: Dr. Ernesto Calderón, Rodríguez Saro N. 523, Department 104-A, Col. Del Valle, C.P. 03100, México D.F., México.

Received 27 December 1996
Accepted 21 April 1997

in test evaluation, sensitivity, specificity and predictive values of the procedures used, and performance time. The prevalence of BV justifies the search for, and design of, a simple low-cost diagnostic method. Since a laboratory test can be more easily standardized than the subjective evaluation of clinical criteria, the following test for the enzymatic activity of proline aminopeptidase (E.C.3.4.11.5) is presented as an alternative diagnostic strategy. This enzyme is normally present in vaginal secretions and rises substantially in BV. Thus, a quick test on a reactive strip was designed to improve on limitations of current laboratory tests.[9,10] The advantages include performance and direct reading in 5 min, and obviates the use of specialized equipment or training in order to confirm a clinical observation in patients with BV.

### MATERIALS AND METHODS

Vaginal secretions were taken from 1,387 outpatients who attended the Clinic of Gynecological Infections at the Hospital Juárez de México from March 1994 to March 1996. A detailed clinical history was also taken from each patient. The questions asked stressed any complaint related to the genitourinary tract, specifically concerning the presence of an unusual amount of vaginal discharge, discomfort due to irritation, itching, bad odor, pain, or bleeding. Clinical and paraclinical criteria for considering positive or negative results for BV as established by Amsel et al.,[2] Thomason et al.,[3] Schoonmaker et al.,[10] and Spiegel et al.[11] were followed.

A routine vaginal examination was performed by means of a disposable speculum without previous lubrication. During the speculumscopy, presence of discharge in the vaginal canal was noted and samples were obtained by rubbing the middle part of the lateral wall of the vagina with sterile cotton swabs.

A swab was rotated on a glass cover. The sample was allowed to dry at room temperature to be Gram-stained and read to evaluate the presence of bacterial morphotypes, as well as the presence of "clue" cells. Another swab saturated with vaginal secretion was discharged by pressing it against the wall of a tube containing 1 ml of saline solution at 0.9%. With this material, homogenized by manual shaking, a glass cover was prepared for direct observation under the microscope, so as to identify the presence of mobile forms compatible with *Trichomonas vaginalis* and *Candida* yeast forms (pseudohyphae). The pH was measured by a reactive strip (using paper with phenaphthazine). For the amine test, a swab with vaginal secretion was submerged into a tube with 1 ml of 10% potassium hydroxide. It was shaken and, if positive, a characteristic fishy odor released from the swab was detected immediately.

The reading and interpretation of the Gram-stained preparation was performed by one of the researchers (R.R.) without knowing the patient's clinical data. The result was considered positive for BV according to 2 criteria: 1) five or fewer morphotypes of *Lactobacillus* observed per field (×1,000); 2) "clue" cells for at least 1 out of every 10 epithelial cells observed (×400): presence of *G. vaginalis* morphotypes, gram-positive coccus, and gram-negative bacillus suggestive of *Bacteroides* and *Mobiluncus* (×1,000).

A sterile swab with vaginal secretion was transported to the laboratory in a tube with 2 ml of brain-heart infusion broth (BHI, Bioxon, Mexico City). The swab was discharged in a semiselective HBT (human blood bilayer tween; BBL Mexico City) medium. The plates were incubated at 37°C under $CO_2$ conditions in a candle jar. The colonies of *G. vaginalis* were presumptively identified after 72 h of incubation by a diffuse margin beta-hemolysis zone, as well as observing the Gram stain with the presence of compatible morphotypes. Identification was complemented by a negative catalase test, hyppurate hydrolysis, and starch utilization.

The criteria for considering a case as BV were presence of a homogenous milky vaginal discharge with pH > 4.5, amine production with a positive fishy odor, presence of "clue" cells, decrease of *Lactobacillus* morphotypes, and a predominance of *G. vaginalis, Bacteroides, Mobiluncus,* and gram-positive coccus morphotypes. The recovery of *G. vaginalis* by culture is not a specific indicator of BV; however, for purposes of the study, the isolation of *G. vaginalis* in culture was considered an additional BV test.

Patients with other pathogens like yeasts *(Candida), T. vaginalis,* or pyogenic bacteria were excluded from the study group. Likewise, patients who appeared healthy were considered a comparison control group.

With regard to development of the test to recognize the presence of proline aminopeptidase (L-Pap) enzyme activity, L-proline β-naphthylamide substrate is broken by enzymatic action at the amino acid level, releasing β-naphthylamide as a final product of the reaction. This is a highly carcinogenic compound. To make the reaction evident requires adding a coloring reagent to visualize the final product. In the original Thomason et al.[3] technique, the procedure is performed on a microplate by adding an avid dye for β-naphthylamide, such as GBC fast garnet salt (Sigma Chemical Co., St. Louis, MO). This step was performed after 4 h of incubation at 35°C. The reading was performed after another 5 min of incubation with the dye. A positive test produces a pink-red color and a negative test produces a yellowish amber color.

Recently, Schoonmaker et al.[10] introduced a change in the technique, by using L-proline p-nitroanilide as a substrate that does not release any carcinogenic residue. After 4 h of incubation on microplates, the samples were visually read without having to add another reagent. A lemon-green reaction was considered positive and no change in the clear color of the plate was considered negative.

### Fast Modification of Both Techniques

Whatman No. 3 filter paper (Sanofi Pasteur, Mexico City) was used, cut into 6 x 0.5 cm strips. All of the reagents used were obtained from Sigma Chemical Co. L-proline β-naphthylamide substrate at 0.2% was prepared by dissolving 200 mg in 100 ml of Tris buffer at pH 7.0. Subsequently, 50 μl aliquots were deposited on previously sterilized strips at their tip (1 cm long end). After drying at room temperature, the strips were stored in an amber jar with desiccator, perfectly closed and placed in a refrigerator at 4°C until used. Recently prepared strips are white. Extended storage, as well as continuous opening to extract them individually from the jar, and remaining outside of the freezer produces a faded reddish color.

The L-proline p-nitroanilide substrate was prepared in the same manner and the strips were stored in a labeled amber jar.

As a control of reactive strip procedures, each test was performed on "U" microplates (Dynatech Labs, Alexandria, VA). A 50 μl aliquot of each substrate, namely, L-proline β-naphthylamide[3] or L-proline p-nitroanilide,[10] was deposited in each well. After preparing and covering them, they were stored frozen at −70°C until used. The coloring reagent to reveal the reaction was used only in the L-proline β-naphthylamide substrate with GBC fast garnet salt at 0.015% (p/v) as the basic ingredient in an aqueous solution, by administering 50 μl for each reaction. This reagent can not tolerate light, therefore, it should be protected. The maximum color of a positive reaction is obtained with a recently prepared reagent. This is stable for about a week if preserved between 2 and 8°C.

### Reactive Strip Procedure to Be Used in a Doctor's Office

A sterile cotton swab saturated with vaginal secretion was applied onto the substrate area at the end of the strip by rotating it vigorously. After 5 min, the developer (fast garnet GBC) was added in the case of β-naphthylamide and the reading was performed. The same procedure was performed with the strip containing L-proline p-nitroanilide substrate and the reaction was read after 5 min.

### Microplate Procedure in the Laboratory

Each sterile cotton swab saturated with vaginal secretion was placed in a glass tube containing 1 ml of saline solution at 0.9%. The contents were discharged by shaking and pressing the swab against the wall of the tube. Each sample was stored frozen at −70°C until further process completion. At the moment of using the sample, it was vortexed and centrifuged at 1,000*g* for 5 min in a refrigerated microcentrifuge (Beckman, Palo Alto, CA). The supernatant was discarded, leaving approximately 100 μl in the vial. From this suspension, 50 μl was taken from each sample and deposited in the microplate wells with the different substrates. Each microplate was incubated at 35°C for 4 h, perfectly covered to prevent evaporation.

At the end of the incubation, GBC fast garnet was added to the β-naphthylamide and incubated for an additional 5 min. Finally, each sample was read individually and the results of each one of the procedures performed on microplates and reactive strips were written down. When a discordant result was observed, the test was repeated.

The sensitivity, specificity, and predictive values of the enzymatic test were calculated[12] for the BV population compared with those obtained for the designated control group.

TABLE 1. Results of 1,087 samples examined

| | L-Pap | |
|---|---|---|
| | (+) | (−) |
| BV and positive culture (N = 388) | 360 | 28 |
| BV and negative culture (N = 95) | 83 | 12 |
| Non-BV and positive culture (N = 47) | 6 | 41 |
| Non-BV and negative culture (N = 557) | 29 | 528 |

### RESULTS

A total of 1,387 gynecological outpatients were seen, of which 300 were not included in our study as they had vaginitis caused by *Candida* (168), *T. vaginalis* (64), both pathogens (18), and atrophic vaginitis (50). The study sample consisted of 1,087 patients, of which 483 (44.4%) complied with the clinical and microscopic criteria to be considered patients with BV. Of the patients with BV, only 239 showed an increase in the vaginal discharge, perigenital itching, and fishy odor. The others (244) were asymptomatic. The remaining 604 (55.6%) made up the control group without BV.

The assays to recognize the presence of L-proline aminopeptidase enzymatic activity with the two substrates (L-proline β-naphthylamide and L-proline p-nitroanilide) coincided for more than 95% of the samples (strips vs. plates). Problem cases due to differences were repeated again. Six swabs were collected from each patient, and it may be possible that numerous vaginal swabs could be responsible for some of the discordant test results (3–5%) due to sampling error. The results that were identical, both in microplate and on reactive strips, were accepted at the end.

According to the results obtained, 4 groups were formed as shown in Table 1.

When the proline aminopeptidase test was positive, the probability of BV (positive predictive value) was 92.6%. The negative predictive value was 93.4%. Likewise, the sensitivity of the reactive strip was 91.7% and the specificity was 94.2% (Table 2).

In 12 of 168 patients (7.1%) with vaginitis caused by *Candida*, the proline aminopeptidase test was positive. The culture was positive for *G. vaginalis* in 9 of these patients. Furthermore, BV diagnosis by Gram stain was positive in those 12 patients.

Reactive strips stored in refrigeration were as efficient as freshly prepared ones, and they remained stable for at least up to 6 months after they were prepared.

TABLE 2. Diagnostic comparison of clinical BV and Gram stain vs. L-proline aminopeptidase

| | Clinical signs and Gram stain | | |
|---|---|---|---|
| Method | BV | Non-BV | Total |
| Proline aminopeptidase | | | |
| Positive | 443 | 35 | 478 |
| Negative | 40 | 569 | 609 |
| Total | 483 | 604 | 1,087 |

### DISCUSSION

A significant percentage of women who have BV are asymptomatic. Thus, if symptoms are not elicited during the examination the syndrome may be undiagnosed. In this study, 244 of 483 (50.5%) women with BV were asymptomatic.

The BV diagnosis is generally based on the presence of three or more aspects observed in the vaginal discharge: white, milky, thin discharge with a homogenous aspect, pH > 4.5, presence of "clue" cells, and a fishy smell of the secretion after adding potassium hydroxide at 10%. The most useful aspects of the pH measurement are its high predictive negative value, as well as its simplicity of performance on a reactive strip that has six colors for pH comparison from 4.0 to 7.0, and obviously its cost effectiveness. However, pH variations can appear in some circumstances such as different stages of the menstrual cycle, menstruation itself, recent sexual intercourse, the use of vaginal douches, or excess cervical mucus.

"Clue" cells require an observer's experience for characterization and accurate definition of the cellular edges occupied by attached bacteria. It is possible to confuse these cells with degenerated cell residues, thickly granulated cells, or even cornified epithelial cells.[4,8,13] The recognition of "clue" cells in direct preparations or Gram-stained smears is a good indicator of BV,[5] however, the procedure is subject to variations that are contingent upon microscopic quality, sample quality, as well as the skill and experience of the observer to perform a careful microscopic examination.

One very common characteristic of BV and trichomoniasis is the fishy odor.[14] This smell is related to the presence of amines (cadaverine, putrescine, methylamine, trimethylamine, etc.). Its presence can vary due to similar circumstances to those observed for measuring vaginal pH. Chen et al.[15] recognized the presence of one or two of these amines in 87% of women with BV, compared to 14% of women without BV; however, in 11 of 63 women with vaginitis caused by mycosis, the test was positive for diamines. The pure *G. vaginalis* culture does not release amines when potassium hydroxide is added thereto.[16] The specific organism responsible for producing amines is unknown, while the most accepted opinion is that it is caused by the combination of anaerobe flora associated with BV.

The criteria of Spiegel et al.[11] for interpreting the presence of the major bacterial morphotypes including *G. vaginalis, Lactobacillus* spp., *Bacteroides* spp., and *Mobiluncus* spp. have been improved by other researchers.[17–19] The reliability of these criteria has demonstrated in recent studies[4,13] that from 62% to 97% of women with BV show consistency between the Gram stain and the clinical diagnosis. On the other hand, 79–95% of women without BV coincide with a Gram stain interpretation as normal flora (predominantly *Lactobacillus*).

The vaginal discharge culture is a procedure available to recover *G. vaginalis* and other morphotypes that are pointed out as responsible for the change of flora in patients with BV. The culture can be considered to have a high sensitivity in women who have clinical BV. Bacteria are recovered from 83% to 94% of these patients,[2,5,13] however, the specificity is low, since the organism can be recovered from 36% to 55% of women who do not have BV symptomatology.

There is no doubt that a simple, diagnostic procedure is currently required that can be reproduced as such when applied during the vaginal exploration.

Proline aminopeptidase is produced by many bacterial species,[20,21] including two BV morphotypes like *G. vaginalis* and *Mobiluncus* spp. During the modification of vaginal flora leading to BV, there is a noticeable increase in the concentration of the enzyme[10] that can be recognized by various procedures.

Aminopeptidase has a specific activity profile that can be used to identify bacteria.[20,21] This procedure can be reproduced through several systems similar to those used by Thomason et al.[3] or Schoonmaker et al.[10] to recognize the presence of enzymatic activity. Aminopeptidase breaks the link between the amino acid and the substrate (proline β-naphthylamide or proline p-nitroanilide), and the residue is avid to reacting to many anilide or nitrate compounds.

The study by Thomason et al.[3] demonstrated how useful it is to analyze enzyme activity on microplates compared to liquid gas chromatography. Sensitivity was 54% for chromatography and 81% for proline aminopeptidase. Specificity for both tests was comparable, namely, 93.6% for chromatography and 96.0% for L-Pap. The authors concluded that the L-Pap assay is superior to liquid gas chromatography, and is an easy method for BV diagnosis that does not require sophisticated equipment, nor special training to perform the test. One limitation is the type of substrate used, i.e., L-proline β-naphthylamide, which, upon breaking due to enzymatic action, leaves β-naphthylamide as the final product with a high carcinogenic risk.

The study by Schoonmaker et al.[10] compared the usefulness of a new substrate, i.e., L-proline p-nitroanilide, which eliminates the disadvantages of L-proline β-naphthylamide. At the same time, it demonstrated the colorimetric quantification of the reaction in a single stage, by using the leucine aminopeptidase microsome as a standard enzyme. Using the criteria of Spiegel et al.[11] to compare both procedures, they found that the sensitivity for tests with a different substrate was similar (93%), while specificity was from 91% to 93%, the positive predictive value was from 78% to 82%, and the negative predictive value was from 97% to 98%. It was concluded then that the procedure using the L-proline p-nitroanilide substrate improved the analysis of the L-Pap activity. Furthermore, it was fast, sensitive, and practical for BV diagnosis.

The design of the reactive strip in this report with either substrate provides for reducing laboratory technology to the maximum, but preferably using L-proline p-nitroanilide as the substrate on the reactive strips. These can be stored desiccated, in refrigeration for long periods without any apparent change in their activity.

Additionally, patients were studied strictly in accordance with clinical criteria and microscopic findings (presence of bacterial morphotypes and "clue cells"). The sensitivity of the reactive strip, which was not influenced by the type of substrate, was 91.7%, specificity was 94.2%, and the probability that a patient having BV had a negative L-Pap test was 6.5% (negative predictive value 93.4%).

On the basis of the results obtained, it is possible to highlight several positive aspects of this fast procedure: 1) it can be directly applied by a doctor or a nurse during the vaginal examination (in the case of the anilide substrate, a developer is not required for the reaction, compared to β-naphthylamide, which requires adding the fast garnet GBC reagent); 2) the reaction on the strip can be directly read in 3–5 min, therefore eliminating incubation time; 3) sensitivity and specificity of the strip are reliable upon accurately selecting the patients and the proper taking of the sample to be placed on the area of the strip containing the substrate; 4) the negative predictive value provides for ensuring that only a small number of ill patients will be incorrectly diagnosed and untreated; 5) the test is quite simple, easy to operate, available in the doctor's office, and cost effective. Therefore, it represents a useful clinical aid procedure in the diagnosis of BV.

### REFERENCES

1. Catlin BW: *Gardnerella vaginalis:* Characteristics, clinical considerations and controversies. Clin Microbiol Rev 5: 213–237, 1992.
2. Amsel R, Totten P, Spiegel CA, Chen KCS, Eschenbach D, Holmes KK: Nonspecific vaginitis: Diagnostic criteria and microbial and epidemiologic associations. Am J Med 74:14–22, 1983.
3. Thomason JL, Schreckenberg PC, Spellacy WN, Riff LJ, LeBeau LJ: Clinical and microbiological characterization of patients with nonspecific vaginosis associated with motile curved anaerobic rods. J Infect Dis 149:801–809, 1984.
4. Thomason JL, Gelbart SM, Anderson RJ, Walt AK, Osypowski MLT, Broekhuizen FF: Statistical evaluation of diagnostic criteria for bacterial vaginosis. Am J Obstet Gynecol 162:155–160, 1990.
5. Eschenbach DA, Hillier SL, Critchlow C, Stevens C, DeRouen T, Holmes KK: Diagnosis and clinical manifestations of bacterial vaginosis. Am J Obstet Gynecol 158:819–828, 1988.
6. Hill GB: The microbiology of bacterial vaginosis. Am J Obstet Gynecol 169:450–454, 1993.
7. Mead PB: Epidemiology of bacterial vaginosis. Am J Obstet Gynecol 169:446–449, 1993.
8. Hillier SL: Diagnostic microbiology of bacterial vaginosis. Am J Obstet Gynecol 169:455–459, 1993.
9. Thomason JL, Gelbart SM, Wilcoski LM, Peterson AK, Jilly BJ, Hamilton PR: Proline aminopeptidase activity as rapid diagnostic test to confirm bacterial vaginosis. Obstet Gynecol 71:607–611, 1988.
10. Schoonmaker JN, Lunt BD, Lawellin DW, French JI, Hillier SL, McGregor JA: A new proline aminopeptidase assay for diagnosis of bacterial vaginosis. Am J Obstet Gynecol 165:737–742, 1991.
11. Spiegel CA, Amsel R, Holmes KK: Diagnosis of bacterial vaginosis by direct Gram stain of vaginal fluid. J Clin Microbiol 18:170–177, 1983.
12. Sox HC: Probability theory in the use of diagnostic test. Ann Intern Med 104:66–73, 1986.
13. Krohn MA, Hillier SL, Eschenbach DA: Comparison of methods for diagnosing bacterial vaginosis among pregnant women. J Clin Microbiol 27:1266–1271, 1989.
14. Clay JC: The odour of non-specific vaginosis: A review. Eur J Clin Microbiol 1:317–319, 1982.
15. Cheng KCS, Amsel R, Eschenbach DA, Holmes KK: Biochemical diagnosis of vaginitis: Determination of diamine in vaginal fluid. J Infect Dis 145:337–345, 1982.
16. Brand JM, Galask RP: Trimethylamine: The substance mainly responsible for the fishy odor often associated with bacterial vaginosis. Obstet Gynecol 63:682–685, 1986.
17. Hay PE, Taylor-Robinson D, Lamont RF: Diagnosis of bacterial vaginosis in a gynaecology clinic. Br J Obstet Gynaecol 99:63–66, 1992.
18. Mazzulli T, Simor AE, Low DE: Reproducibility of a scoring system for Gram stain diagnosis of bacterial vaginosis. J Clin Microbiol 29:1730–1731, 1991.
19. Nugent RP, Krohn MJ, Hillier SL: Reliability of diagnosing bacterial vaginosis is improved by a standardized method of Gram stain interpretation. J Clin Microbiol 29:297–301, 1991.
20. Westley JW, Anderson PJ, Close VA, Halpern B, Lederberg EM: Aminopeptidase profiles of various bacteria. Appl Microbiol 15:822–825, 1967.
21. Watson RR: Substrate specificities of aminopeptidases. A specific method for microbial differentiation. In Norris JR (ed): Methods in Microbiology. Vol 9. New York: Academic Press, pp 1–14, 1976.
